# Supplementary material for: A loss-of-adhesion CRISPR-Cas9 screening platform to identify cell adhesion-regulatory proteins and signaling pathways
Source: Nat Commun. 2022 Apr 19;13:2136. doi: 10.1038/s41467-022-29835-y (PMC9018714; doi:10.1038/s41467-022-29835-y)
Supplement: Supplementary file 1 — Supplementary Information [file 41467_2022_29835_MOESM1_ESM.pdf]

SUPPLEMENTARY TABLE AND FIGURES

GuideID	BrunelloID	Guide sequence	Gibson cloning oligo for LentiGuide x BsmBI
sgNEG	BRDT-2	ATGGCCCTTTCAACGTCCTG	TATCTTGTGGAAAGGACGAAACACCG**ATGGCCCTTTCAACGTCCTG**GTTTTAGAGCTAGAAATAGCAAGTTAAAA
sgNT	NT-1	AAAAAGCTTCCGCCTGATGG	TATCTTGTGGAAAGGACGAAACACCG**AAAAAGCTTCCGCCTGATGG**GTTTTAGAGCTAGAAATAGCAAGTTAAAA
sgBTK-A	BTK-2	CTGTGTTTGCTAAATCCACA	TATCTTGTGGAAAGGACGAAACACCG**CTGTGTTTGCTAAATCCACA**GTTTTAGAGCTAGAAATAGCAAGTTAAAA
sgBTK-B	BTK-3	GATGGTAGTTAATGAGCTCA	TATCTTGTGGAAAGGACGAAACACCG**GATGGTAGTTAATGAGCTCA**GTTTTAGAGCTAGAAATAGCAAGTTAAAA
sgPAK2-A	PAK2-5	CTAGGAACTATCGTGAGCAT	TATCTTGTGGAAAGGACGAAACACCG**CTAGGAACTATCGTGAGCAT**GTTTTAGAGCTAGAAATAGCAAGTTAAAA
sgPAK2-B	PAK2-8	TGACGTTGCACTGGGACAGG	TATCTTGTGGAAAGGACGAAACACCG**TGACGTTGCACTGGGACAGG**GTTTTAGAGCTAGAAATAGCAAGTTAAAA
sgPKM-A	PKM-6	GCTGTGGCTCTAGACACTAA	TATCTTGTGGAAAGGACGAAACACCG**GCTGTGGCTCTAGACACTAA**GTTTTAGAGCTAGAAATAGCAAGTTAAAA
sgPKM-B	PKM-7	TCACTGCCTTCAGCCCGAGT	TATCTTGTGGAAAGGACGAAACACCG**TCACTGCCTTCAGCCCGAGT**GTTTTAGAGCTAGAAATAGCAAGTTAAAA
sgCRKL-A	CRKL-5	ACCGCTCCGCCTGGTATATG	TATCTTGTGGAAAGGACGAAACACCG**ACCGCTCCGCCTGGTATATG**GTTTTAGAGCTAGAAATAGCAAGTTAAAA
sgCRKL-B	CRKL-6	CCTGGGCGCAGGCTCGATGA	TATCTTGTGGAAAGGACGAAACACCG**CCTGGGCGCAGGCTCGATGA**GTTTTAGAGCTAGAAATAGCAAGTTAAAA
sgPTK2B-A	PTK2B-6	CTGGGGACATCCATTTGATG	TATCTTGTGGAAAGGACGAAACACCG**CTGGGGACATCCATTTGATG**GTTTTAGAGCTAGAAATAGCAAGTTAAAA
sgPTK2B-B	PTK2B-7	GCGGAACAAGAACTCCCTGA	TATCTTGTGGAAAGGACGAAACACCG**GCGGAACAAGAACTCCCTGA**GTTTTAGAGCTAGAAATAGCAAGTTAAAA

Supplementary table 1. CRISPRguide sequences.

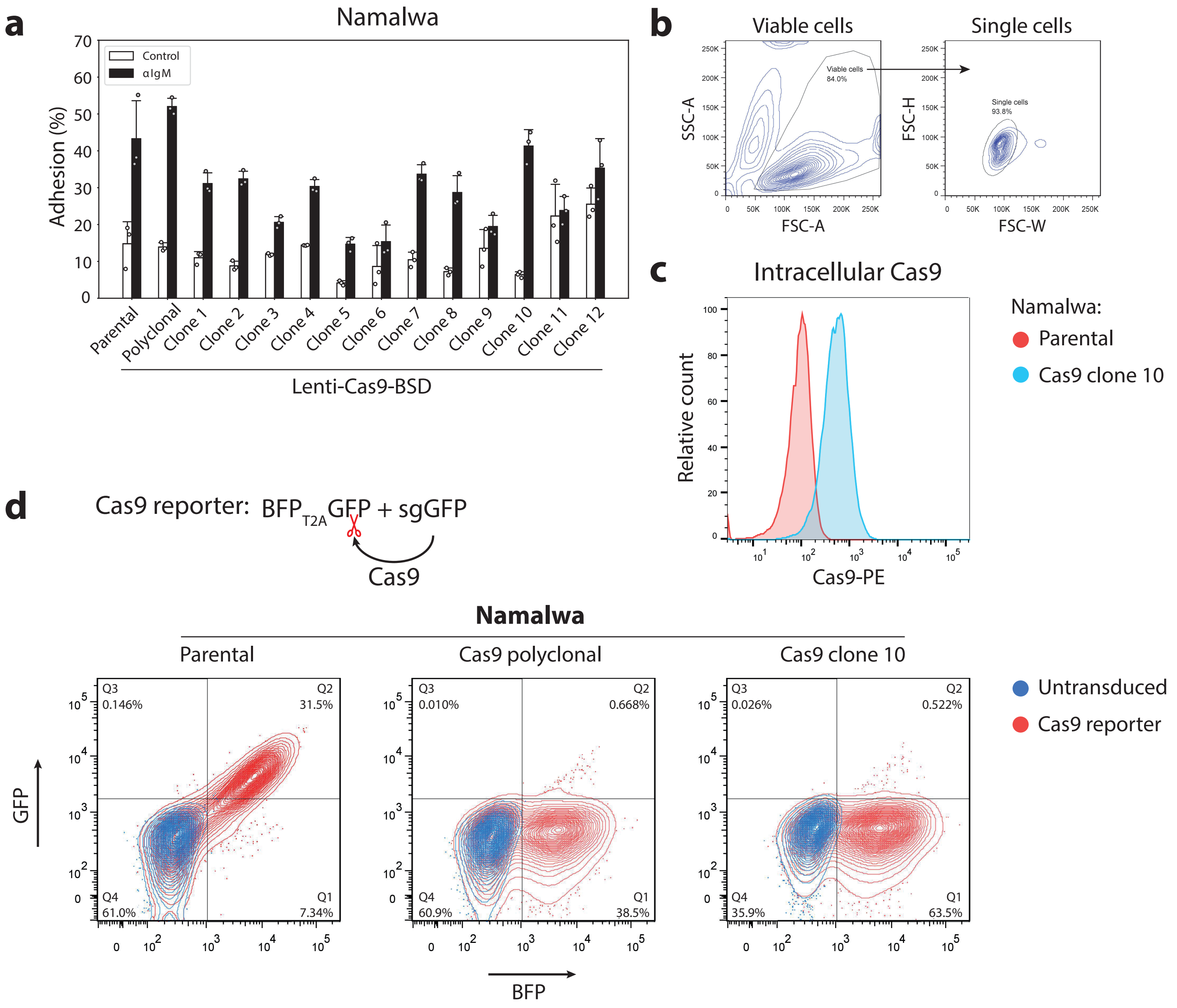

Supplementary figure 1. Generation of Cas9 clones. a Namalwa cells were transduced with Lenti-Cas9-BSD, blasticidin-selected, and sorted 1 cell/well. Growing clones were first tested for αIgM-induced adhesion to fibronectin. The clone with the best stimulated/unstimulated ratio was selected for screening (clone 10). Graph is presented as mean + SD of triplicates. The clone selection was performed once. Source data are provided as a Source Data file. **b** Gating strategy to select viable single cells for FACS analysis. **c** Clone 10 was tested for Cas9 expression by intracellular FACS. Cas9 expression was analyzed once. **d** Clone 10 was tested for Cas9 activity with the Cas9-reporter. Cas9 activity results in loss of GFP, but not BFP expression. Cas9 activity was regularly tested, and we observed no loss of Cas9 activity, even after prolonged culturing in the absence of blasticidin.

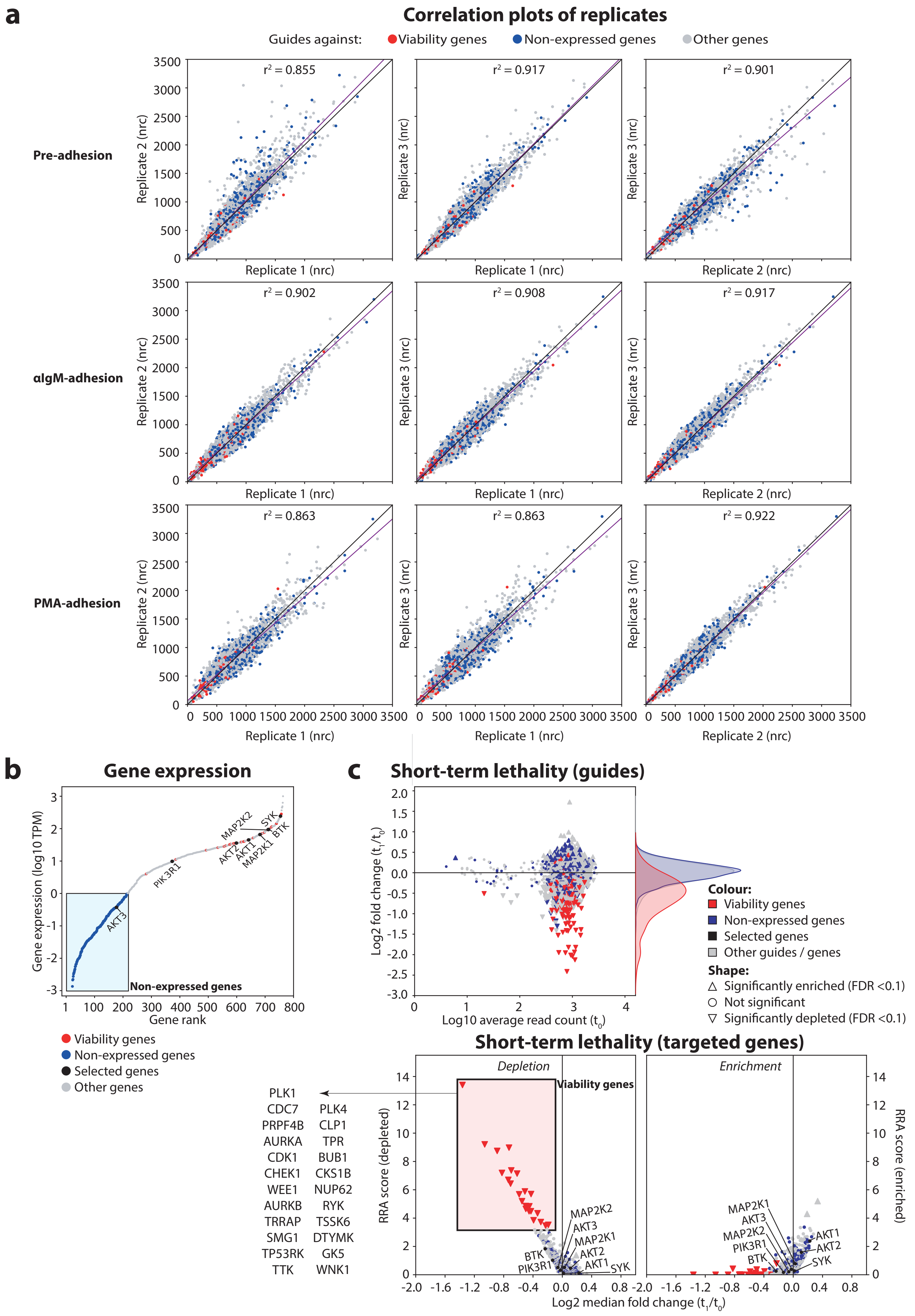
a
Correlation plots of replicates
Guides against: Viability genes Non-expressed genes Other genes
Pre-adhesion
αIgM-adhesion
PMA-adhesion
r² = 0.855
r² = 0.917
r² = 0.901
r² = 0.902
r² = 0.908
r² = 0.917
r² = 0.863
r² = 0.863
r² = 0.922
Replicate 2 (nrc)
Replicate 3 (nrc)
Replicate 1 (nrc)
Replicate 2 (nrc)
0 500 1000 1500 2000 2500 3000 3500
b
Gene expression
Gene expression (log10 TPM)
Gene rank
MAP2K2
SYK
BTK
AKT2
AKT1
MAP2K1
PIK3R1
AKT3
Non-expressed genes
Viability genes
Non-expressed genes
Selected genes
Other genes
c
Short-term lethality (guides)
Log2 fold change (t1/t0)
Log10 average read count (t0)
Colour:
Viability genes
Non-expressed genes
Selected genes
Other guides / genes
Shape:
Significantly enriched (FDR <0.1)
Not significant
Significantly depleted (FDR <0.1)
Short-term lethality (targeted genes)
Depletion
Enrichment
Viability genes
RRA score (depleted)
RRA score (enriched)
Log2 median fold change (t1/t0)
PLK1
CDC7 PLK4
PRPF4B CLP1
AURKA TPR
CDK1 BUB1
CHEK1 CKS1B
WEE1 NUP62
AURKB RYK
TRRAP TSSK6
SMG1 DTYMK
TP53RK GK5
TTK WNK1
MAP2K2
AKT3
MAP2K1
AKT2
AKT1
SYK
BTK
PIK3R1
MAP2K1
AKT3
MAP2K2
PIK3R1
BTK
AKT1
AKT2
SYK

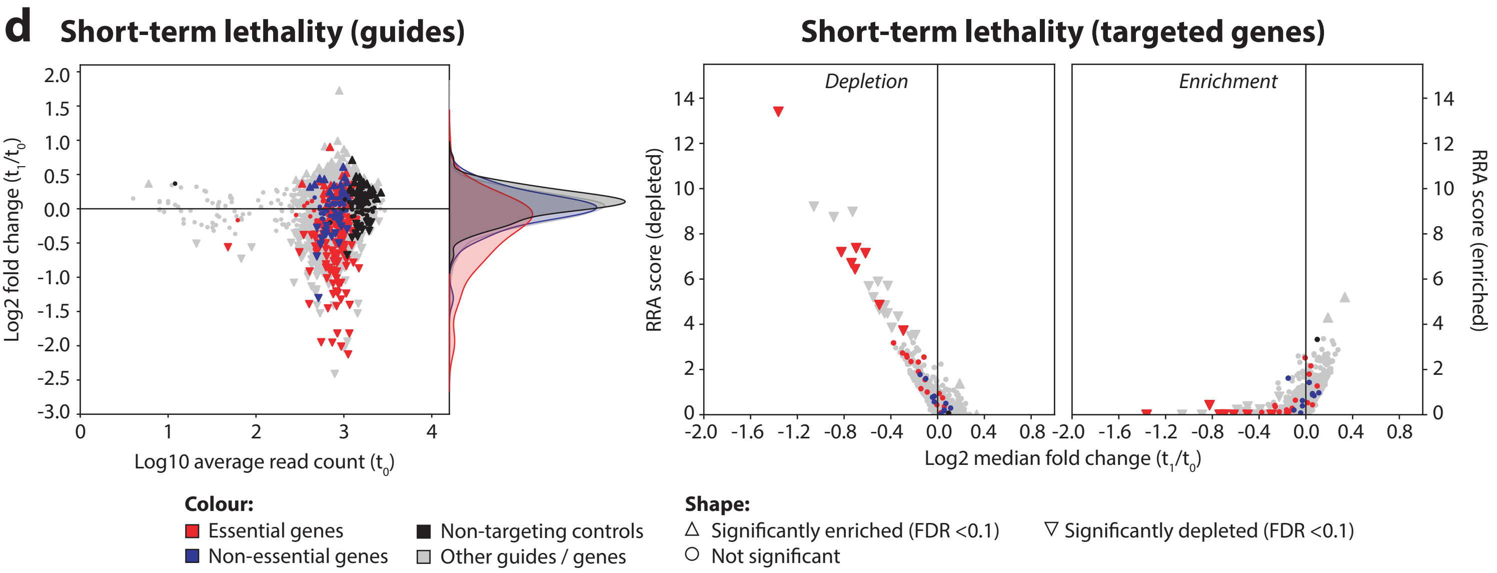

Supplementary figure 2. Replicate plots of the adhesion screen and definition of non-expressed and viability genes. a Replicate plots of guide distribution in pre-adhesion, αIgM-induced adhesion, and PMA-induced adhesion samples. nrc: normalized read counts, black line y=x, purple line: linear fit. **b** Genes represented in the Brunello kinome-centered library were selected from public Namalwa RNAseq data, and ranked by gene expression. Genes which express less than 1 transcript per million (TPM) were defined as non-expressed genes in Namalwa. Of note, the non-essential/non-expressed gene BRDT had an expression level of 0.0 TPM (not shown). **c** MA and volcano plots of pre-adhesion samples (t_1 = 8 days upon library transduction) compared to the library distribution (t_0). The genes in which the corresponding guides dropped out significantly (FDR <0.1, DESeq2 followed by $\alpha RRA_{depletion}$) were defined as (strong) viability genes. The screen was performed with 3 independent replicates. **d** The same MA and volcano plots as in c, but here the guides against essential and non-essential genes (described by Hart *et al*[31]) and non-targeting control guides are depicted. Source data are provided as a Source Data file.

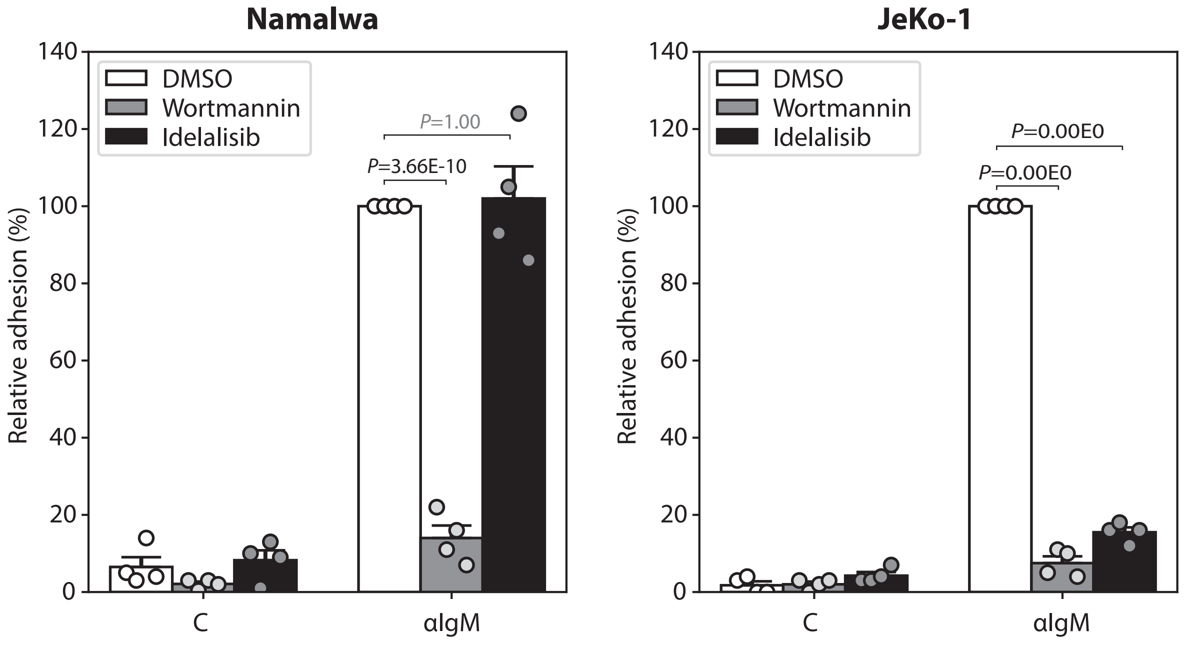

Supplementary figure 3. PI3Kδ is not essential for BCR-controlled adhesion of Namalwa cells. Namalwa and JeKo-1 cells pretreated with 100nM wortmannin (pan-PI3K inhibitor), 1µM idelalisib (PI3Kδ inhibitor), or DMSO control, were stimulated with αIgM, and allowed to adhere to fibronectin-coated surfaces. Graphs are presented as normalized means + SEM (100% = untreated αIgM-stimulated cells). *P*-values are adjusted *P*-values from two-way ANOVA followed by Tukey HSD post hoc test (two-tailed and paired design), of 4 independent experiments each assayed in triplicate. Source data are provided as a Source Data file.

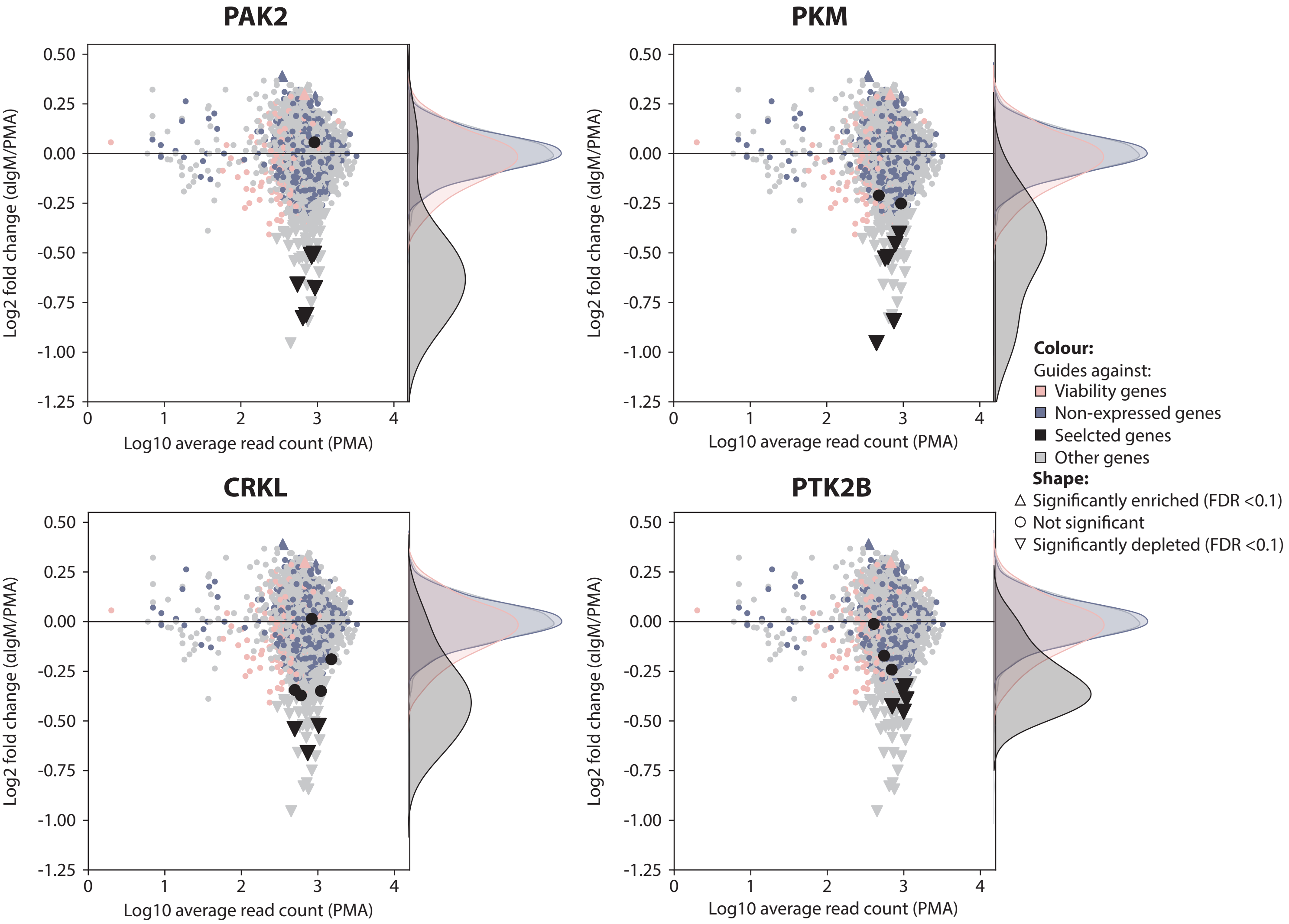

Supplementary figure 4. MA plots of αIgM-induced adhesion relative to PMA-induced adhesion of the novel regulators of proximal BCR-integrin signaling. Statistics was performed with DESeq2. The screen was performed with 3 independent replicates. Source data are provided as a Source Data file.

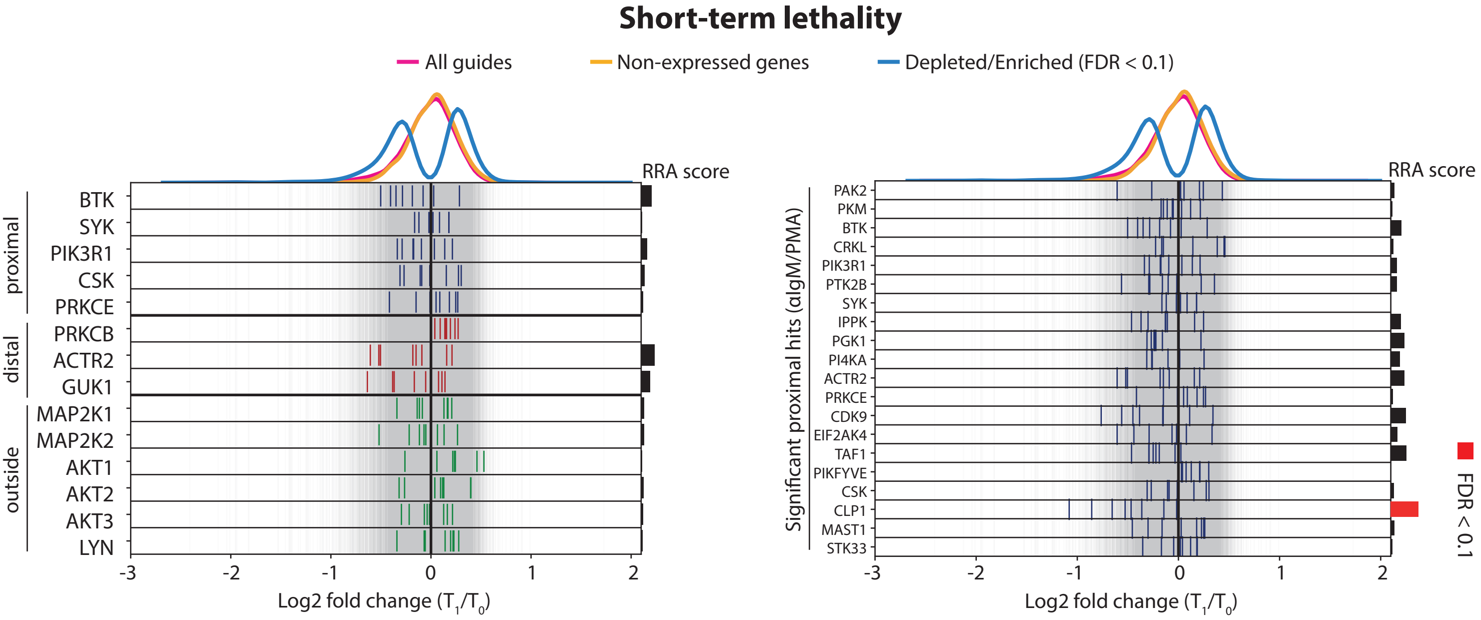

Supplementary figure 5. Analysis of the short-term lethality of the guides targeting genes involved in the antigen/BCR-integrin axis. Extension of figures 3 (left) and 4a (right), showing the effect on short-term lethality of the guides against BCR signaling components. Statistics was performed with DESeq2 followed by αRRA. The RRA scores are $\alpha RRA_{depletion}$ scores. The screen was performed with 3 independent replicates. Source data are provided as a Source Data file.

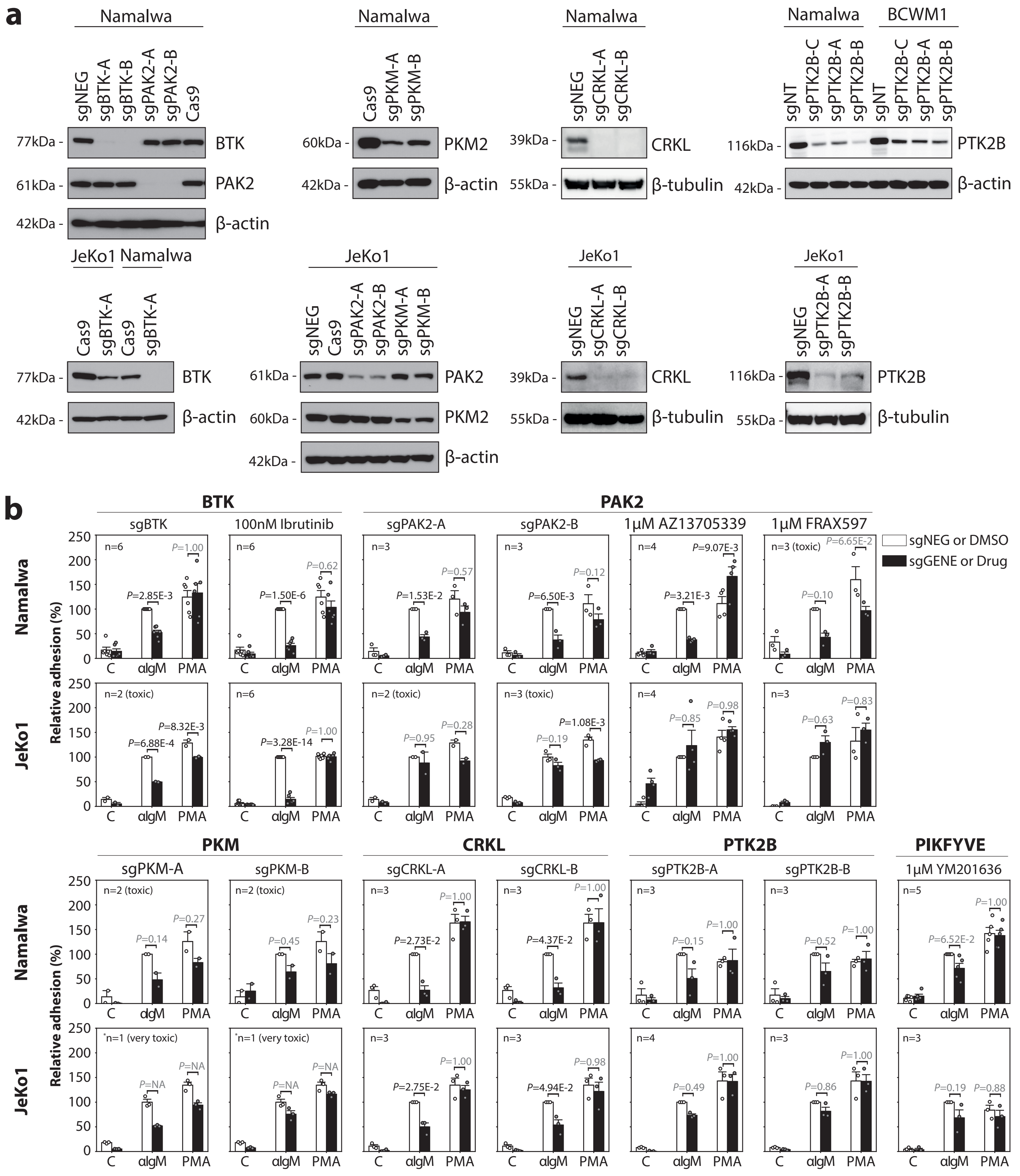

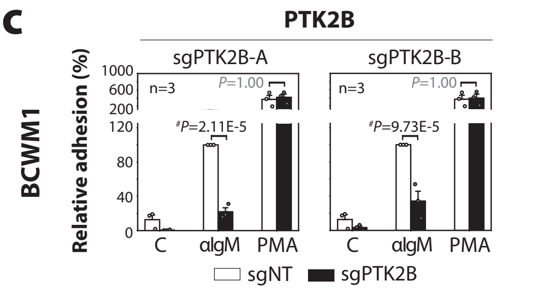

Supplementary figure 6. Validation of the novel proximal BCR-signaling hits. **a** CRISPR knock outs of BTK, PAK2, PKM, CRKL, and PTK2B in Namalwa and JeKo1 cells, and PTK2B in BCWM1 cells were verified by western blot. A guide against the nonessential/nonexpressed gene BRDT (sgNEG) or a nontargeting guide (sgNT) were used as control guides. Cas9 represents the parental cells. Knockout upon each transduction was at least two times verified by western blot. **b-c** Namalwa, JeKo1, and BCWM1 cells with indicated knock out or 1h pretreated with indicated drug, were stimulated with αIgM or PMA, and allowed to adhere to fibronectin-coated surfaces. Graphs are presented as normalized means + SEM (100% = untreated αIgM-stimulated cells). *P*-values are adjusted *P*-values from two-way ANOVA followed by Tukey HSD post hoc test (two-tailed and paired design), of n independent experiments each assayed in triplicate. *Technical replicates are shown. #PMA values were excluded in the statistics, because they were in another range than the αIgM values. Source data are provided as a Source Data file.
